# Exploring Immunohistochemistry in Fish: Assessment of Antibody Reactivity by Western Immunoblotting

**DOI:** 10.3390/ani13182934

**Published:** 2023-09-15

**Authors:** Elisabetta Antuofermo, Massimo Orioles, Claudio Murgia, Giovanni P. Burrai, Martina Penati, Chiara Gottardi, Marta Polinas, Donatella Volpatti, Marco Galeotti, Maria Filippa Addis

**Affiliations:** 1 Dipartimento di Medicina Veterinaria, Università degli Studi di Sassari, Via Vienna 2, 07100 Sassari, Italy; eantuofermo@uniss.it (E.A.); c.murgia3@studenti.uniss.it (C.M.); mpolinas@uniss.it (M.P.); 2 Veterinary Pathology Unit, Department of Agricultural, Food, Environmental and Animal Sciences, University of Udine, 33100 Udine, Italy; massimo.orioles@uniud.it (M.O.); donatella.volpatti@uniud.it (D.V.); marco.galeotti@uniud.it (M.G.); 3 Dipartimento di Medicina Veterinaria e Scienze Animali, Università degli Studi di Milano, Via dell’Università 6, 26900 Lodi, Italy; martina.penati@unimi.it (M.P.); chiara.gottardi@studenti.unimi.it (C.G.); filippa.addis@unimi.it (M.F.A.)

**Keywords:** antibody validation, aquaculture, cytokeratin, vimentin, S-100, GFAP, desmin, fish pathology, immunohistochemistry

## Abstract

**Simple Summary:**

In recent years, fish research has seen significant advancements, driven by the expansion of aquaculture species production, the ornamental fish industry, and biomedical studies involving aquatic organisms. Immunohistochemistry (IHC) has emerged as a valuable tool in veterinary research for studying fish biology and pathology. However, the need for validated antibodies optimized for fish species remains a challenge, leading to potential false results and misinterpretations. This study systematically assessed the reactivity of commercially available antibodies (CK AE1/AE3, vimentin, S-100, GFAP, and desmin) in IHC assays on four fish species: *Sparus aurata*, *Dicentrarchus labrax*, *Oncorhynchus mykiss*, and *Carassius auratus*. We employed Western immunoblotting (WB) and IHC techniques to evaluate antibody specificity. The results revealed a good cross-reactivity for anti-cytokeratin AE1/AE3, GFAP, and S-100 antibodies, demonstrating specific staining. Conversely, vimentin and desmin antibodies displayed no reactivity. In conclusion, this research emphasizes the need for validating antibodies specifically for fish species to ensure accurate and reliable results in fish research involving IHC analysis.

**Abstract:**

In recent years, research on fish has seen remarkable advancements, especially in aquaculture, ornamental fish industry, and biomedical studies. Immunohistochemistry has become crucial in fish research, aiding in physiological and pathological investigations. However, the use of antibodies originally developed for mammals has raised concerns about their cross-reactivity and specificity in fish. This study systematically evaluated the reactivity of commonly used antibodies for diagnostic purposes, especially in fish pathology, including pan-cytokeratin, vimentin, S-100, glial fibrillary acidic protein, and desmin in the tissue of *Sparus aurata*, *Dicentrarchus labrax*, *Oncorhynchus mykiss*, and *Carassius auratus*. Western immunoblotting was employed to assess antibody specificity. The results revealed that the pan-cytokeratin and glial fibrillary acidic protein antibodies cross-react with all tested fish species, while S-100 demonstrated specific staining in sea bream, goldfish, and rainbow trout tissues. Conversely, vimentin and desmin antibodies displayed no reactivity. In conclusion, the anti-cytokeratin clone AE1/AE3 and the polyclonal rabbit anti-glial fibrillary acidic protein antibody, which are extensively used in mammals, were validated for fish immunohistochemical studies. Regrettably, D33 anti-desmin and V9 anti-vimentin clones are unsuitable for immunohistochemistry in the tested fish. These findings underscore the need for species-specific antibodies and proper validation for accurate immunohistochemistry analyses in fish research.

## 1. Introduction

In recent years, research on fish has made unprecedented steps forward, propelled by the increase in aquaculture species production, the ornamental fish industry, and biomedical studies involving aquatic organisms. Despite notable advancements, several challenges persist. One prominent issue revolves around the vast array of fish species and their phylogenetic diversity, each characterized by distinct biology and physiology. Furthermore, diagnosing fish diseases presents a complex undertaking, requiring precise tools and methodologies specifically designed and validated for aquatic animals.

Among the different assays, immunohistochemistry (IHC) is an effective technique that has rapidly gained popularity in veterinary research, finding applications in physiological and pathological studies [1]. IHC has emerged as a valuable microscopy-based method in anatomic, physiological, and pathological fish studies [2,3,4,5,6,7], specifically in identifying infectious disease agents and characterizing neoplastic lesions [8]. Except for zebrafish (*Danio rerio*), researchers often rely on antibodies developed against human or mouse proteins, commonly lacking species-specificity. Despite their extensive use [9,10,11,12,13,14,15,16], mammalian antibodies must be optimized for aquatic animals [17,18]. Thus, false positives, negatives, and aberrant labelling are commonly described, despite manufacturers’ data sheets providing some information regarding expected cross-species reactivity.

Antibody cross-reactivity in IHC refers to the ability of an antibody to bind to the same antigen in different species or when the antibody is designed to recognize a conserved epitope on the antigen [1]. In aquatic animals, an antibody that reacts effectively in one species may not exhibit similar performance in others [17,18]. The clonality of antibodies represents another confounding element related to IHC, as polyclonal antibodies may show cross-reactivity with common epitopes expressed by different proteins, leading to a non-specific background. Finally, interspecies variations in antibody reactions stem from changes in the amino acid sequence of antigens, leading to different reactivity among species, even for identical antibody clones targeting the same antigen. It is, therefore, essential to validate the IHC cross-reactivity of antibodies with the corresponding antigen in the fish tissue species of interest using specific methods. Among the many approaches to validate the IHC assay’s specificity, the Western blotting (WB) technique is the preferred choice for evaluating cross-reactivity, allowing the detection of the protein’s molecular weight and determining the specificity of detection [19,20].

Antibodies frequently used for IHC with variable results in fish research are cytokeratins, vimentin, glial fibrillary acidic protein (GFAP), S-100, and desmin. Cytokeratin antibodies are used to detect a group of cytoplasmic proteins playing a role in maintaining cellular integrity, serving as markers for epithelial tissues. The expression of the 20 existent different cytokeratins is complex as their pattern has been restricted during evolution [2] and can change during a lifetime [3]. Pan-cytokeratin AE1/AE3 antibodies are commonly employed to identify and characterize epithelial cells in various fish species [2,3,18,21], and positive reactions were found in teleost skin, intestine, renal tubules, certain glia, and thymic epithelial cells [3,7,21]. In pathological studies, neoplastic tissues such as carcinomas, adenocarcinomas, and papillomas were characterized by using cytokeratin antibodies [9,22,23].

As an intermediate filament protein, vimentin exhibits widespread cytoplasmic expression in mesenchymal cell types, including fibroblasts, endothelial cells, melanocytes, and smooth muscle cells [24]. However, vimentin staining in normal teleost tissues and neoplastic disease has been reported with variable positivity [5,15,21,22].

The S-100 protein, belonging to the EF-hand superfamily, plays an essential role in cell proliferation, differentiation, apoptosis, gene transcription, and intracellular calcium homeostasis [25]. In teleosts, S-100 used to detect calcium-binding proteins is a moderately non-specific marker capable of staining cells of neurocrest origin, nerve fibers, melanocytes, sensory organs, brain, spinal cord [4,26,27], and neoplasia such as schwannoma [5,28].

GFAP, a protein belonging to the family of intermediate filaments, plays a crucial role in providing structural support to astrocytes and maintaining the integrity of the central nervous system (CNS). GFAP is associated with various neurological disorders such as brain injury, inflammation, and neurodegenerative diseases, or as a marker of neoplasms originating from glial cells in the CNS. GFAP expression is used in combination with other markers and histological features to classify gliomas into various subtypes, such as astrocytomas, oligodendrogliomas, and glioblastomas in mammals [29]. In fish, GFAP has been used to map glial structures both in the brain and other areas such as the gut [30,31], as well as a tumor marker [32].

Desmin is a type of intermediate filament protein that is an essential component of skeletal and cardiac muscle, maintaining the integrity of muscle fibers. In IHC, antibodies against desmin are used as specific markers for muscle tissue and are often utilized to differentiate muscle-related neoplasms, such as rhabdomyosarcoma, from other types of tumors [29]. Desmin antibodies have been employed to identify muscle cells in teleost species, making them helpful in investigating muscle-related diseases and disorders [33,34].

This study aims to assess the cross-reactivity of commercially available antibodies (CK AE1/AE3, vimentin, S-100, GFAP, and desmin) in IHC assays conducted on *Sparus aurata*, *Dicentrarchus labrax*, *Oncorhynchus mykiss*, and *Carassius auratus*. By systematically testing and validating these antibodies by WB, we ascertain their specificity, sensitivity, and reproducibility across various commonly encountered teleost fish tissues.

## 2. Materials and Methods

### 2.1. Sample Preparation and Histology

Paraffin-embedded and frozen (at −80 °C) tissues of the skin, brain, heart, and intestine from a total of 12 fish were included in this study. In particular, fish were selected as follows: three farmed gilthead sea bream (*Sparus aurata*—Mediterranean seabream, adult fish, ranging from 30 to 38 cm in length and between 350 and 450 g in weight), three European sea bass (*Dicentrarchus labrax*—Western Mediterranean seabass, adult fish, ranging from 35 to 42 cm in length and between 300 and 450 g in weight); three goldfish (*Carassius auratus*—Ryukin strain, adult fish, ranging from 10 to 15 cm in length and weight between 20 and 25 g) and three rainbow trout (*Oncorhynchus mykiss*—Danish strain, adult fish, ranging from 52 to 55 cm in length and weight between 250 and 350 g from the diagnostic archives of Sassari and Udine Universities, respectively.

Before inclusion in paraffin, all the tissues were previously fixed in 10% buffered formalin for 48 h, dehydrated with increasing alcohol concentrations and xylene in an automatic tissue processor. Sections of 3 µm thickness were obtained with a microtome (RM2245, Leica Biosystems, Wetzlar, Germany) and stained with hematoxylin and eosin in an automatic multi-stainer (ST5020, Leica Biosystems, Wetzlar, Germany). The inclusion criteria for this study were: the absence of gross and microscopically significant lesions and the presence of known antigen expression in tissue. Canine tissues from archive diagnostic material (brain, heart, intestine, skin) were included as a positive control. Experiment permission was not required from the University’s Animal Care Ethics Committee since all the samples were used for diagnostic purposes.

### 2.2. Antibodies and Sequence Alignments

The following antibodies were used for the study: Dako, monoclonal mouse anti-cytokeratin CKAE1/AE3; Dako, monoclonal mouse anti-vimentin, clone V9; Dako, polyclonal rabbit anti-S100, code Z0311; Dako, polyclonal rabbit anti-glial fibrillary acidic protein, code Z0334; Dako, mouse monoclonal anti-human desmin, clone D33. For all the antibodies providing sufficient information, the UniProt entry sequence of the antigen was retrieved and aligned with the respective protein sequences of *S. aurata*, *D. labrax*, *C. auratus*, and *O. mykiss*. When more than one sequence was available, the one with the highest annotation level was selected for the alignment; when available, the UniProtKB reviewed sequence was used. Protein sequence alignments were carried out with the Align tool of the Universal Protein KnowledgeBase (https://www.uniprot.org/, accessed on 24 august 2023).

### 2.3. SDS–PAGE and Western Immunoblotting

Fresh-frozen aliquots of fish tissues were minced with a sterile scalpel. Then, 100 mg of each tissue was resuspended in 500 µL of Laemmli Buffer (Sigma-Aldrich, St. Louis, MO, USA) and incubated at 100 °C for 10 min at 1500 rpm in a Thermomixer Comfort (Eppendorf, Hamburg, Germany). All extracts were clarified for 5 min at 10,000× *g* at 4 °C and separated by sodium dodecyl sulfate–polyacrylamide gel electrophoresis (SDS–PAGE) in AnykD^TM^ polyacrylamide gels on a Protean Tetra Cell (Bio-Rad, Hercules, CA, USA) following manufacturer’s instructions. After the run, gels were stained with SimplyBlue™ Protein SafeStain (Thermo Fisher Scientific, Waltham, MA, USA).

For WB, separated proteins were transferred to nitrocellulose membranes with the Trans-Blot^®^ Turbo^TM^ Transfer System (Bio-Rad). Nitrocellulose membranes were checked for quality by reversible Ponceau S staining (Sigma-Aldrich), destained with water, blocked with EveryBlot Blocking Buffer (Bio-Rad) for 5 min and then incubated for 1 h with the following primary antibodies diluted in EveryBlot Blocking Buffer: anti-cytokeratin, dilution 1:1000, anti-vimentin, dilution 1:1000, anti-S-100, dilution 1:5000, glial fibrillary acidic protein (GFAP), dilution 1:5000, and desmin, dilution 1:1000. The membranes were then washed five times with PBS-T (phosphate buffered saline, 0.05% Tween 20) and incubated with appropriate HRP-conjugated anti-mouse antibodies or with HRP-conjugated anti-rabbit antibodies (Sigma-Aldrich) diluted in blocking buffer (1:2000) for 30 min. After five washes with PBS-T, the reactivity was visualized with a chemiluminescent peroxidase substrate (Clarity Western ECL substrate, Bio-Rad). Chemiluminescent images were digitalized with an iBright 1500 (Thermo Fisher Scientific). Molecular weight (MW) was estimated using the MagicMark markers (Thermo Fisher Scientific).

### 2.4. Immunohistochemistry

Serial sections of skin, brain, heart, and intestine were mounted on positively charged slides (Superfrost, Fisher Scientific) for immunostaining. Slides were immersed for 20 min in a 98 °C, preheated solution (WCAP, citrate pH 6, BiOptica, Milan, Italy) for antigen unmasking. Tissues were blocked for endogenous peroxidase (Dako REAL Peroxidase-Blocking Solution S2023, Dako, Glostrup, Denmark) and non-specific binding with 2.5% normal horse serum (ImmPRESS reagent kit, Vector Labs, Burlingame, CA, USA) and 2% bovine serum albumin (BSA) Sections were incubated overnight at 4 °C with the same primary antibodies as reported above at the following dilutions: anti-cytokeratin AE1/AE3, 1:200, anti-vimentin, 1:200, S-100, 1: 2000, GFAP, 1:2000, anti-desmin, 1:200. Then, the sections were incubated with an anti-mouse/rabbit secondary antibody (ImmPRESS reagent kit—peroxidase—MP-7500; Vector Laboratories, Burlingame, CA, USA) for 30 min at room temperature and treated with 3,30 -Diaminobenzidine (DAB) chromogen (ImmPACT DAB; Vector Laboratories). Tissues were then counterstained with hematoxylin, dehydrated, and mounted. Slides were evaluated under light microscopy (Nikon Eclipse 80i) and digital computer images were recorded with a Nikon Ds-fi1 camera. Normal canine tissue, including skin, brain, heart, and intestine were used as positive controls. Negative controls were established by replacing the primary antibodies with only antibody diluent.

## 3. Results

### 3.1. Antibodies and Sequence Alignments

The protein homology matrices and the sequence alignment results for all the antigens with an available sequence are reported in the Supplementary File S1. The pan-cytokeratin antibody could not be assessed as the antigen is an uncharacterized protein mixture from a human callus extract; therefore, protein sequence information is not available. Concerning the other proteins, the vimentin antigen displayed a sequence homology ranging from 76.4% for C. auratus to 74.34% for *O. mykiss*. The sequence homology for the S100 antigen ranged from 33.33% for *C. auratus* to 62.77% for *D. labrax*. The sequence homology for the GFAP antigen ranged from 33.10% for *S. aurata* to 72.05% for *C. auratus*. Finally, the sequence homology of desmin ranged from 37.18% for *C. auratus* to 74.16% for *D. labrax*. 

### 3.2. Pan-Cytokeratin

**Figure 1:**
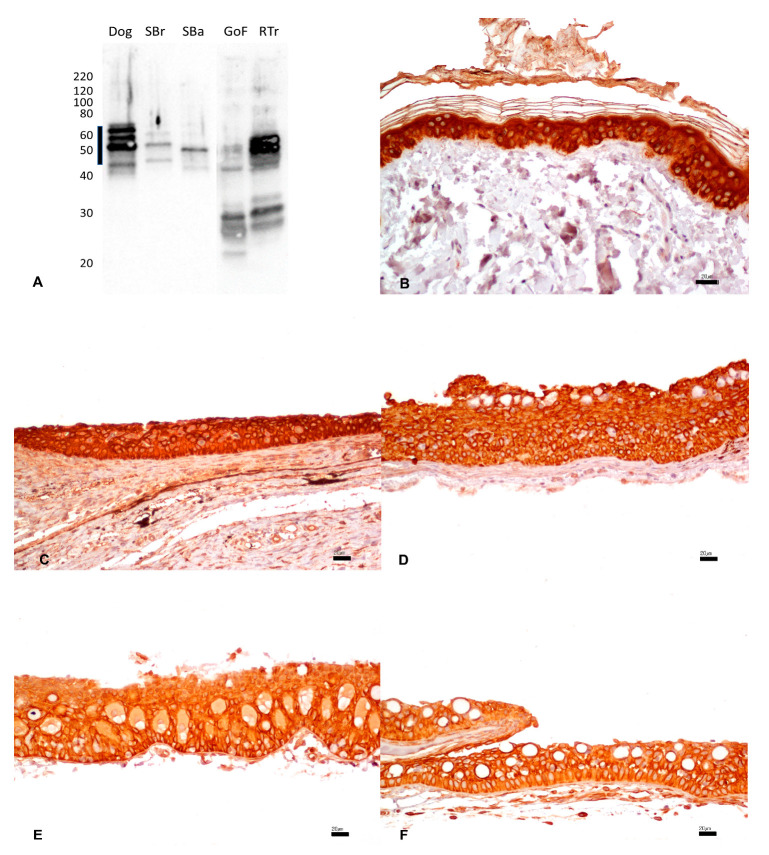
(**A**) Western immunoblotting of skin tissues incubated with the monoclonal anti-pan-cytokeratin antibody. Molecular weight markers are indicated on the left. The predicted molecular weight range of 45–65 kDa is indicated with a thick line. Dog tissue extract loaded as a positive control; SBr, sea bream; SBa, sea bass; GoF, goldfish; RTr, rainbow trout. (**A**–**F**). IHC shows strong and diffuse cytoplasmic immunostaining with accentuation of the cellular membrane in the squamous epithelium of the skin in the dog (**B**), sea bream (**C**), sea bass (**D**), goldfish (**E**), and rainbow trout (**F**). Bar: 20 µm.

The reactivity of mouse monoclonal anti-human cytokeratin AE1/AE3 antibodies was assessed by WB against skin tissue extracts. Several clear bands within the predicted molecular mass range of 45–65 kDa were observed in all the species tested (Figure 1A). In goldfish and rainbow trout, lower molecular weight bands in the range of 30 kDa were also observed, indicating possible additional non-specific reactivity of the tested antibody in these two species.

By IHC, a strong and diffuse cytoplasmic immunostaining highlighting the cellular membrane in the squamous epithelium of the skin was observed both in the dog (Figure 1B) and all fish species (Figure 1C–F), using the same monoclonal antibody (Table 1). 

### 3.3. Vimentin

**Figure 2:**
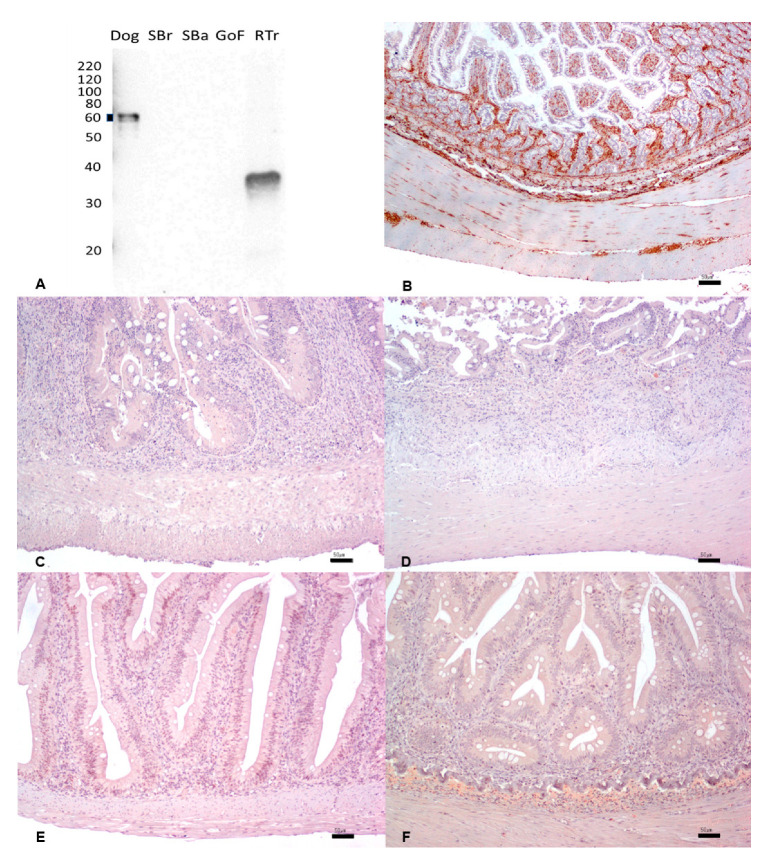
(**A**) Western immunoblotting of intestinal tissues incubated with the mouse monoclonal anti-vimentin antibody. Molecular weight markers are indicated on the left. The predicted molecular weight of 60 kDa is indicated with a thick line. Dog tissue extract loaded as a positive control; SBr, sea bream; SBa, sea bass; GoF, goldfish; RTr, rainbow trout. (**B**–**F**). IHC shows strong and diffuse cytoplasmic immunostaining of the intestinal mesenchymal cells of the dog (**B**), while no immunosignals were observed in sea bream (**C**), sea bass (**D**), goldfish (**E**), and rainbow trout (**F**). Bar: 5.0 µm.

The reactivity of mouse monoclonal anti-vimentin antibody was tested against intestinal tissue extracts. The dog tissue proteins tested as a positive control produced a band at the expected MW of ~60 kDa (Figure 2A). No reactivity was observed for sea bream, sea bass, or goldfish. The rainbow trout extract displayed a band at ~35, indicating possible non-specific reactivity.

By IHC, strong and diffuse cytoplasmic staining was observed with the same anti-vimentin antibody in the mesenchymal cells of the intestine in dog tissues (Figure 2B). No immunostaining was present in fish tissues (Figure 2C–F) (Table 1).

### 3.4. S100 Protein

**Figure 3:**
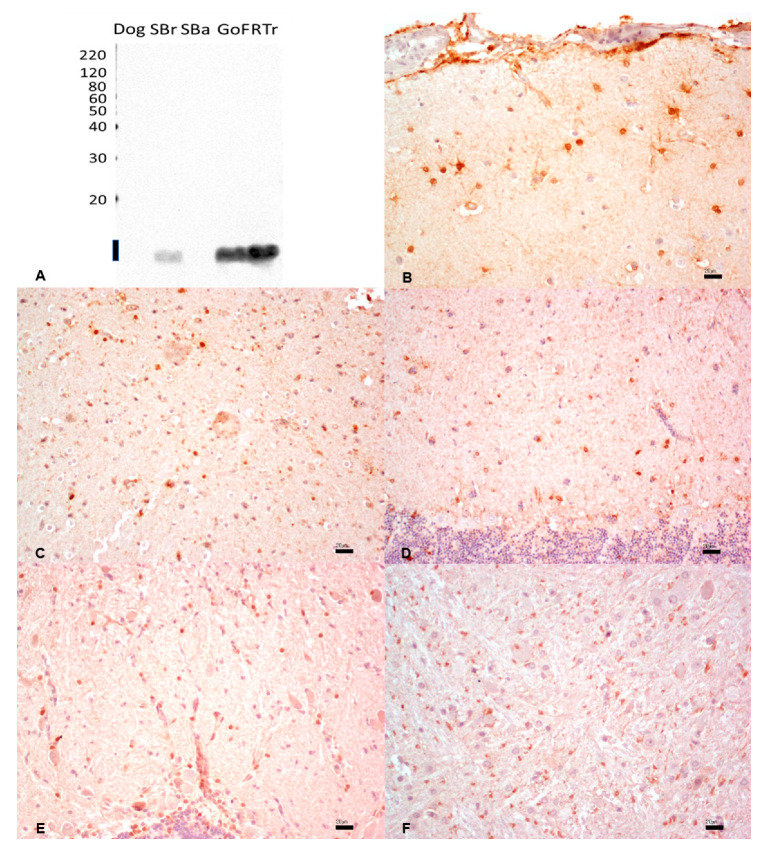
(**A**) Western immunoblotting of brain tissues incubated with the rabbit polyclonal anti-S100 protein. Molecular weight markers are indicated on the left. The predicted molecular weight of 10–12 kDa is indicated with a thick line. Dog tissue extract loaded as a positive control; SBr, sea bream; SBa, sea bass; GoF, goldfish; RTr, rainbow trout. (**B**–**F**). IHC shows a strong and diffuse cytoplasmic and nuclear expression in the dog (**B**), in sea bream (**C**), sea bass (**D**), goldfish (**E**), and rainbow trout (**F**). Bar: 20 µm.

The reactivity of rabbit polyclonal anti-S100 antibodies was assessed against brain tissue extracts. The dog tissue proteins tested as a positive control did not produce any band, indicating a lack of reactivity of the antibody in this species. The same was observed for sea bass proteins. On the other hand, the antibody produced a faint band in sea bream tissues and a very intense band in goldfish and rainbow trout at ~10 kDa. In these species, the findings shown in Figure 3A indicate the likelihood of specific S100 protein isoforms being recognized by the antibody (Figure 3A).

In contrast, IHC demonstrated a strong and diffuse nuclear and cytoplasmic expression of S100 protein in all species’ cytoplasm, and in nuclei in the brain tissues. (Figure 3B–F) (Table 1).

### 3.5. Glial Fibrillary Acidic Protein

**Figure 4:**
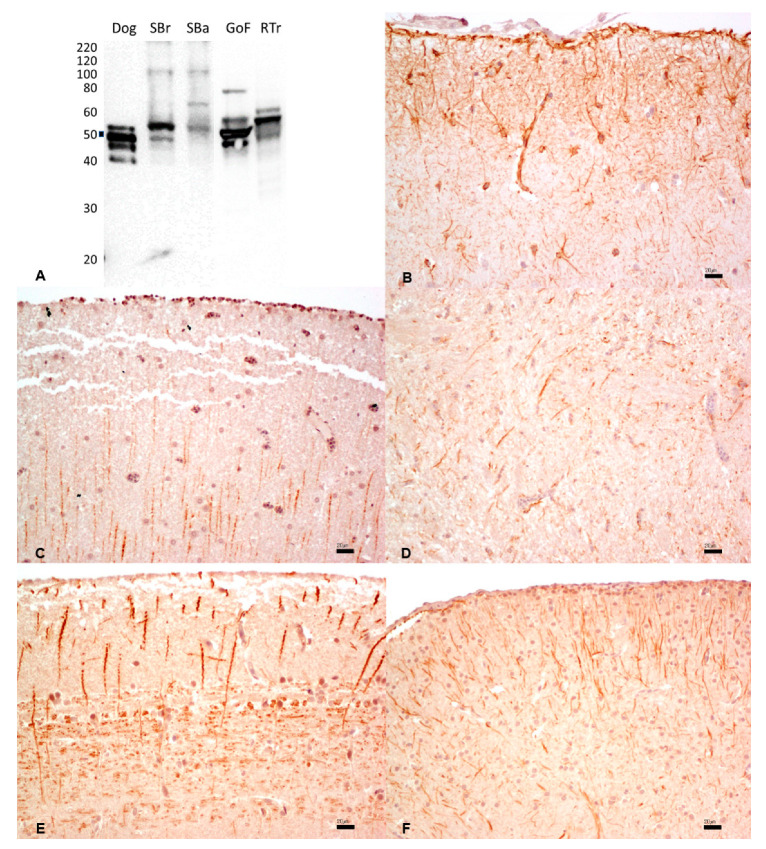
(**A**) Western immunoblotting of brain tissues incubated with the rabbit polyclonal anti-glial fibrillary acidic protein (GFAP). Molecular weight markers are indicated on the left. The predicted molecular weight of 50 kDa is indicated with a thick line. Dog tissue extract loaded as a positive control; SBr, sea bream; SBa, sea bass; GoF, goldfish; RTr, rainbow trout. (**B**–**F**). IHC shows strong and diffuse cytoplasmic staining in the dog (**B**), sea bream (**C**), sea bass (**D**), goldfish (**E**), and rainbow trout (**F**). Bar: 20 µm.

The reactivity of rabbit polyclonal anti-GFAP was tested against brain tissue extracts. The dog tissue proteins tested as a positive control produced a band at the expected MW of ~50 kDa plus other minor bands, possibly due to multiple protein isoforms. Bands of similar MW were observed in all the fish species tested, although with minor differences, which might be related to species-specific isoforms. Higher molecular weight bands observed in sea bream and sea bass (~100 kDa) may represent protein dimers (Figure 4A).

The immunostaining performed by IHC with the same anti-GFAP antibodies produced a strong and diffuse cytoplasmic staining of glial cells (i.e., astrocytes) in all tested species (Figure 4B–F) (Table 1).

### 3.6. Desmin

**Figure 5:**
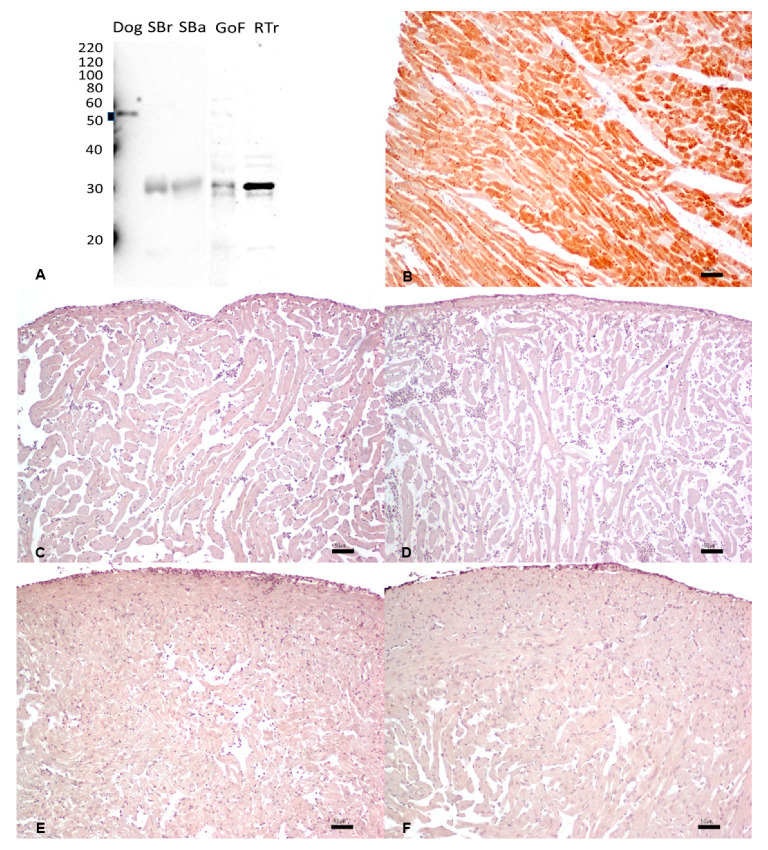
(**A**) Western immunoblotting of cardiac tissues incubated with the monoclonal anti-human desmin antibody. Molecular weight markers are indicated on the left. The predicted molecular weight of ~50 kDa is indicated with a thick line. Dog tissue extract loaded as a positive control; SBr, sea bream; SBa, sea bass; GoF, goldfish; RTr, rainbow trout. (**B**–**F**). By IHC, a strong and diffuse cytoplasmic expression was observed in the dog (**B**), while no immunostaining was observed in sea bream (**C**), sea bass (**D**), goldfish (**E**), and rainbow trout (**F**). Bar: 50 µm.

The reactivity of the mouse monoclonal anti-human desmin antibody was assessed against heart tissue extracts. The dog tissue proteins tested as a positive control produced a band at the expected MW of ~50 kDa. Fish tissues displayed a band in the 30 kDa molecular weight, indicating possible non-specific reactivity (Figure 5A). By IHC, the same anti-desmin antibody did not show any reactivity in the cardiac tissues of all examined fishes (Figure 5C–F). Conversely, a strong signal was observed in dog myocardium (Figure 5B) (Table 1).

**Table 1:** Summary of the results obtained by Western immunoblotting (WB) and immunohistochemistry (IHC) with all the assessed antibodies and tissues. The (+) symbol indicates antibody reactivity, whereas the (-) symbol the absence of antibody reactivity, and (×) possible non-specific reactivity.

Antibody and Tissue	Species	WB	IHC
Pan-cytokeratin (skin) Monoclonal mouse anti-human cytokeratin CKAE1/AE3	Canine	+	+
	*Sparus aurata*	+	+
	*Dicentrarchus labrax*	+	+
	*Carassius auratus*	+	+
	*Oncorhynchus mykiss*	+	+
Vimentin (intestine) Monoclonal mouse anti-vimentin, clone V9	Canine	+	+
	*Sparus aurata*	-	-
	*Dicentrarchus labrax*	-	-
	*Carassius auratus*	-	-
	*Oncorhynchus mykiss*	×	-
S-100 (brain) Polyclonal rabbit, code Z0311	Canine	-	+
	*Sparus aurata*	+	+
	*Dicentrarchus labrax*	-	+
	*Carassius auratus*	+	+
	*Oncorhynchus mykiss*	+	+
GFAP (brain) Polyclonal rabbit anti-glial fibrillary acidic protein, code Z0334	Canine	+	+
	*Sparus aurata*	+	+
	*Dicentrarchus labrax*	+	+
	*Carassius auratus*	+	+
	*Oncorhynchus mykiss*	+	+
Desmin (skin) Mouse monoclonal anti-human desmin, clone D33	Canine	+	+
	*Sparus aurata*	×	-
	*Dicentrarchus labrax*	×	-
	*Carassius auratus*	×	-
	*Oncorhynchus mykiss*	×	-

## 4. Discussion

Immunohistochemistry is a fundamental technique widely employed in biomedical research and diagnostic pathology, constituting a potent tool that provides crucial insights into protein expression, antigens cellular localization, and disease-related alterations within tissues. Its applications extend across various fields, including veterinary science, where it significantly advances our understanding of the biology and pathogenesis of animal diseases.

In aquatic organisms, IHC has found prominent utility in exploring anatomical and pathological aspects, particularly concerning neoplastic disorders in farmed and ornamental fish species [5,7,9,10,11,13,14,17]. Nevertheless, the specificity of antibodies holds paramount importance in ensuring accurate results, and it is worth noting that many commercially available antibodies might have yet to undergo thorough validation for their application in fish tissues. Antibodies such as cytokeratin (AE1/AE3), vimentin, S-100 protein, GFAP, and desmin, primarily developed for human or mouse proteins, may lack the species-specificity for fish. Consequently, the potential for false positives or negatives arises, leading to misinterpretation of results [17]. To address this concern, WB has emerged as a preferred technique for testing antibody cross-reactivity and enhancing the reliability of IHC in fish research [19]. This study investigates the immunolocalization of the most encountered antibodies in teleosts, assessing their specificity through WB to establish their applicability in fish species. 

The clone AE1/AE3 represents a cocktail of antibodies capable of detecting cytokeratins 1–8, 10, 14–16, and 19, with its expression visualized through membrane positivity. Our findings indicate that this clone exhibited cross-reactivity in all tested fish species, producing bands between 52 and 65 kDa, corresponding to the predicted molecular weight range. Notably, it demonstrated a robust cytoplasmic staining reaction, focusing on the epithelial cells of the species under investigation. These results are consistent with those revealed by previous IHC studies in fish, highlighting the role played by the monoclonal mouse anti-CK antibody in recognizing cytokeratins in epithelial cells of diverse fish species, both in healthy tissues and neoplasms [2,3,5,7,9,18,21,23]. The results strongly suggest that the clone AEl/AE3 antibody exhibits significant cross-reactivity between mammals and fish proteins. Hence, it can be considered a viable option for conducting immunohistochemical studies in tested fish species.

In mammals, vimentin is a common immunohistochemical marker for distinguishing between epithelial and mesenchymal tissues and identifying tumors exhibiting a mesenchymal phenotype, such as sarcomas [35]. In fish pathology, the V9 mouse monoclonal antibody is widely used for immunohistochemical analysis to identify normal and neoplastic mesenchymal cells with variable results [5,10,12,36]. The results of the present study show that the V9 clone does not effectively bind to its intended target protein in tested fish tissues, except for a non-specific WB-detectable band in rainbow trout. Given the protein homology between the antigen used for antibody generation and the fish proteins (>70% for all species), the observed lack of reactivity may be attributed to other factors, including crucial sequence differences in the epitope region, potential cross-reactivity issues, or variations in protein expression levels in the investigated fish tissues. These findings are in line with recent studies in which the V9 clone failed to cross-react with goldfish peripheral nerve sheath tumors (atypical neurofibroma) [36]. However, our results differ from prior reports wherein vimentin exhibited a positive reaction in adult ovarian cells and a gonadal tumor in koi carp (SCST) [21], as well as in the outer layers (fibroblasts) of granulomas developed against histozoic metazoan parasites in mullet [16]. Moreover, our results do not agree with what was reported by Šálková and coauthors [7], as they successfully utilized the anti-vimentin antibody (clone V9) in both sterlet and carp without conducting a validation test such as WB. Based on the findings of this study, it is reasonable to conclude that the monoclonal mouse anti-human vimentin clone V9 is unsuitable for immunohistochemical studies in the examined fish species.

S-100 is another commonly used marker in IHC studies for identifying normal cells, tumors, and diseases originating from the neural crest in fish: its effectiveness as an IHC marker shows slight variation across different fish species [4,7,26,27]. Our study confirms this data in IHC, showing a strong and diffuse cytoplasmic and nuclear expression of S-100 in the brain of all tested species. However, WB results showed the presence of bands at approximately 10 kDa for sea bream, goldfish, and rainbow trout tissues. Our WB results closely aligned with other researchers who reported bands using polyclonal antibodies against the S100 protein, revealing a molecular weight of 10 kDa [4,25]. Interestingly, a weaker band was observed in sea bream, and no bands were observed in sea bass, suggesting a lack of antibody reactivity in this latter species. When examining the percent identity matrix for this antigen, the sea bass showed the highest homology (62.77%). On the other hand, the strongest reactivity was observed for goldfish, for which the percent homology between antigen and target protein was the lowest (33.33%). Likely, the sequence differences occurring in the epitope play a vital role in the antibody’s ability to recognize the fish protein variants. Unfortunately, the epitope sequences are not available. Additionally, no band was detected in the dog brain extracts; further investigation would be advisable, considering the extensive use of this marker in veterinary medicine. Given these considerations, the polyclonal rabbit S-100 antibody may constitute a useful tool when considering the species-specific diversity in fish.

GFAP antibodies facilitate the differential diagnosis of peripheral nervous sheet tumors such as Schwannomas and neurofibromas in fish [29,33,36]. Our results showed that the polyclonal anti-rabbit GFAP antibody displays strong specificity at approximately 50 kDa molecular weight and exhibits cytoplasmic expression in brain glial cells across all tested species, confirming the antibody’s specificity. In this case, Western immunoblotting in sea bream and sea bass observed a slightly weaker reactivity in line with their lower protein sequence homology with the antigen used for antibody generation. Our results align with those highlighted by a previous study, where a monoclonal GFAP anti-mouse antibody was employed, validating the expression of GFAP proteins at 90 kDa and 50–52 kDa in the brain and spinal cord of two larval stages and adult rainbow trout [37]. Therefore, similar to cytokeratin, we can conclude that there is a good cross-reactivity between mammals and fish when using this polyclonal rabbit anti-GFAP antibody, which is now validated for immunohistochemical investigation in fish species.

In contrast to dogs, where a strong signal was detected, no immunostaining reaction of the anti-desmin antibody against the target protein, commonly utilized as a specific marker for muscle tissue and for distinguishing muscle-related neoplasms, was observed in the heart of all the examined fish species. By WB, the anti-desmin mouse monoclonal antibody demonstrated the presence of an expected 53 kDa band in the dog heart. However, no signal was evident at this molecular weight in fish. Additional bands at lower molecular weights were detected in all fish examined, suggesting potential antibody cross-reactivity with different peptides. These results collectively indicate that clone D33 may not be suitable for studying muscle-related tissues in tested fish. This observation is consistent with findings from various other authors [33,36], suggesting that desmin might be a helpful marker only for newly formed or regenerating fibers in fish [33] or should be considered unsuitable for immunohistochemical studies in fish species, as indicated by Armando and coauthors [36]. This discrepancy exists despite a substantial sequence homology with the protein antigen (>70% for sea bass, rainbow trout, and sea bream).

## 5. Conclusions

The results of this study underscore the importance of validating antibodies for IHC assays in the specific species of interest, especially in teleost fish where cross-reactivity with mammalian antibodies can vary significantly. While the tested clones of anti-cytokeratin, GFAP, and S-100 (only for sea bream, goldfish, and rainbow trout) antibodies provided reliable results, other antibodies, such as vimentin and desmin exhibited no cross-reactivity in fish species examined. 

For future studies involving immunohistochemical analysis in fish, it is recommended to carefully select antibodies that have been specifically validated to assess antibody specificity and cross-reactivity in fish tissues. Additionally, species-specific antibodies or those specifically optimized for aquatic animals should be sought to ensure accurate and reproducible results in fish research.

In conclusion, this study provides valuable insights into the performance of commonly used antibodies in fish tissues, highlighting the need for cautious selection and validation of antibodies in aquatic animal studies. By employing appropriate antibodies and optimizing IHC protocols, understanding fish biology, pathology, and disease can advance, contributing to the continued progress in aquaculture, the ornamental fish industry, and biomedical studies involving aquatic organisms.

## Data Availability

Not applicable.

## Supplementary Materials

The following supporting information can be downloaded at: https://www.mdpi.com/article/10.3390/ani13182934/s1, Supplementary File S1: Supplementary file with protein alignments.
