# Supplementary material for: Exploring Immunohistochemistry in Fish: Assessment of Antibody Reactivity by Western Immunoblotting
Source: Animals (Basel). 2023 Sep 15;13(18):2934. doi: 10.3390/ani13182934 (PMC10525475; doi:10.3390/ani13182934)
Supplement: Supplementary file 1 [file animals-13-02934-s001.zip › Supplementary File S1.pdf]

Supplementary File with protein alignments.

A. Percent identity matrices for vimentin, S100-A1, glial fibrillary acidic protein (GFAP), desmin.

B. CLUSTAL O(1.2.4) multiple sequence alignment for vimentin.

C. CLUSTAL O(1.2.4) multiple sequence alignment for S100 A9.

D. CLUSTAL O(1.2.4) multiple sequence alignment for S100 A9.

E. CLUSTAL O(1.2.4) multiple sequence alignment for desmin.

A. Percent identity matrices with the Uniprot Entry of the immunogens provided by the manufacturer.

Vimentin Percent Identity Matrix - created by Clustal2.1

1: sp|P48674|VIME_ONCMY	100.00	74.34	76.33	75.06	76.40
2: sp|P02543|VIME_PIG	74.34	100.00	75.29	71.68	76.40
3: sp|P48671|VIM1_CARAU	76.33	75.29	100.00	80.47	74.32
4: tr|A0A671V9L3|A0A671V9L3_SPAAU	75.06	71.68	80.47	100.00	95.68
5: tr|A0A411KAS9|A0A411KAS9_DICLA	76.40	76.40	74.32	95.68	100.00

S100 Percent Identity Matrix - created by Clustal2.1

1: tr|A0A6P6R5E7|A0A6P6R5E7_CARAU	100.00	28.26	23.91	33.33	25.27
2: tr|A0A671W834|A0A671W834_SPAAU	28.26	100.00	49.49	45.74	46.94
3: tr|C1BH93|C1BH93_ONCMY	23.91	49.49	100.00	52.13	52.04
4: sp|P02639|S10A1_BOVIN	33.33	45.74	52.13	100.00	62.77
5: tr|A0A8C4I043|A0A8C4I043_DICLA	25.27	46.94	52.04	62.77	100.00

GFAP Percent Identity Matrix - created by Clustal2.1

1: tr|A0A671WZT8|A0A671WZT8_SPAAU	100.00	33.10	32.48	31.49	35.26
2: sp|Q28115|GFAP_BOVIN	33.10	100.00	64.37	61.68	72.05
3: tr|A0A8C7NQ31|A0A8C7NQ31_ONCMY	32.48	64.37	100.00	76.57	80.39
4: tr|A0A8P4G1A2|A0A8P4G1A2_DICLA	31.49	61.68	76.57	100.00	82.19
5: sp|P48677|GFAP_CARAU	35.26	72.05	80.39	82.19	100.00

Desmin Percent Identity Matrix - created by Clustal2.1

1: tr|A0A6P6PJ00|A0A6P6PJ00_CARAU	100.00	37.18	37.66	39.75	37.19
2: sp|P17661|DESM_HUMAN	37.18	100.00	74.16	71.95	72.47
3: tr|A0A8C4I534|A0A8C4I534_DICLA	37.66	74.16	100.00	77.96	78.24
4: tr|Q8UWF1|Q8UWF1_ONCMY	39.75	71.95	77.96	100.00	82.18
5: tr|A0A2R2YUL3|A0A2R2YUL3_SPAAU	37.19	72.47	78.24	82.18	100.00

B. CLUSTAL O(1.2.4) multiple sequence alignment for vimentin.

```
sp|P48674|VIME_ONCMY                MNRTTSRQTTSSSSYKRMFGGEGRPSVGMARSTLSSRQYSSPVR-----------SSRMS  49
sp|P02543|VIME_PIG                  ----MSTRTVSSSSYRRMFGGPGTASR----PSSSRSYVTTSTRTYSLGSALRPSTSRSL  52
sp|P48671|VIM1_CARAU                ------------------------------------------------------------  0
tr|A0A671V9L3|A0A671V9L3_SPAAU      ----MSHRATPSSSYRKMFGGERVSAR----SSYTSRQFSIPVR-----------SSRAT  41
tr|A0A411KAS9|A0A411KAS9_DICLA      ------------------------------------------------------------  0

sp|P48674|VIME_ONCMY                YSVSAPPSIYASKN--VRLRSSAPMPRLSSDTVDFALSDAINSEFKANRTNEKAEMQHLN  107
sp|P02543|VIME_PIG                  YT-SSPGGVYATRSSAVRLRSSVPGVRLLQDAVDFSLADAINTEFKNTRTNEKVELQELN  111
sp|P48671|VIM1_CARAU                ------------------------------------------------------------  0
tr|A0A671V9L3|A0A671V9L3_SPAAU      YGVSLAPTVYAAKT--QRLRSTAAMPRLASENLDFSLSDAINSEFMTNRTNEKVQMQSLN  99
tr|A0A411KAS9|A0A411KAS9_DICLA      ------------------------------------------------------------  0

sp|P48674|VIME_ONCMY                DRFASYIDKVRFLEQQNKILLAELEQLKGKGASRIGDLYEDEMRDLRRQVDQLTNEKAHV  167
sp|P02543|VIME_PIG                  DRFANYIDKVRFLEQQNKILLAELEQLKGQGKSRLGDLYEEEMRELRRQVDQLTNDKARV  171
sp|P48671|VIM1_CARAU                ------------------------------------------------------------  0
tr|A0A671V9L3|A0A671V9L3_SPAAU      DRFASYIEKVRFLEQQNKILLAELEQLRGKGTSRVGDLYEDEMRELRRQVDQLTNEKARV  159
tr|A0A411KAS9|A0A411KAS9_DICLA      ------------------------------------------------------------  0

sp|P48674|VIME_ONCMY                EVDRDNMGEDIERLREKLQDEMIQKEEAEHNLQSFRQDVDNASLARLDLERKVESLQEEI  227
sp|P02543|VIME_PIG                  EVERDNLAEDIMRLREKLQEETLQREEAESTLQSFRQDVDNASLARLDLERKVESLQEEI  231
sp|P48671|VIM1_CARAU                ------------------------------------------------------------  0
tr|A0A671V9L3|A0A671V9L3_SPAAU      EVYRDNLAEDIDRLREKLQDEIAQREDAESNMQSFRQDVDNAALARLDLERKVESLQDEI  219
tr|A0A411KAS9|A0A411KAS9_DICLA      --------------------------------------VDNAALARLDLERKVESLQDEI  22

sp|P48674|VIME_ONCMY                IFLRKLHDEEVAELQAQIQ-DQHVQIDMDVAKPDLTAALRDVRVQYETLASRNLQDSEDW  286
sp|P02543|VIME_PIG                  AFLKKLHDEEIQELQAQIQ-EQHVQIDMDVSKPDLTAALRDVRQQYESVAAKNLQEAEEW  290
sp|P48671|VIM1_CARAU                ------------------------------------------------------------  0
tr|A0A671V9L3|A0A671V9L3_SPAAU      NFLKKLHDEEMLELQSQMQQQQHVQVDMEMAKPDLTAALRDVRLQYENLASKNIHESEEW  279
tr|A0A411KAS9|A0A411KAS9_DICLA      NFLKKLHDEEMLELQNQMQQQQHVQVDMEMAKPDLTAALRDVRLQYENLASKNIHESEEW  82

sp|P48674|VIME_ONCMY                YKSKFADLSEAANRNTDAIRQAKQEANEYRRQVQALTCEVDSLKGTNESMERQMRELEES  346
sp|P02543|VIME_PIG                  YKSKFADLSEAANRNNDALRQAKQESNEYRRQVQSLTCEVDALKGTNESLERQMREMEEN  350
sp|P48671|VIM1_CARAU                ------DLTEAANKSNEALRLAKQESNDYRRQVQALTCEVDALKGTNESLERQMREMEEN  54
tr|A0A671V9L3|A0A671V9L3_SPAAU      YKSKFADLTEAAARNNDALRVAKQEANDYRRQVQALTCEVDALKGTNESLDRQMREMEEN  339
tr|A0A411KAS9|A0A411KAS9_DICLA      YKSKFADLTEAAARNNDALRVAKQEANDYRRQVQALTCEVDALKGTNESMERQIRELEEN  142
                                          **:*** :..:*:* ****:*:******:******:*******::**:**:**.

sp|P48674|VIME_ONCMY                FGCEANNFQDTISRLEDDIRNMKDEMARHLREYQDLLNVKMALDIEIATYRKLLEGEESR  406
sp|P02543|VIME_PIG                  FAVEAANYQDTIGRLQDEIQNMKEEMARHLREYQDLLNVKMALDIEIATYRKLLEGEESR  410
sp|P48671|VIM1_CARAU                FAMESSSSQDKIVQLEEDTQNMKDEMAKHLHEYQDLLNVKMALDIEIATYRKLLEGEESR  114
tr|A0A671V9L3|A0A671V9L3_SPAAU      FSLETGGYQDTIGRLEEDIHNMKDEMARHLREYQDLLNVKMALDIEIATYRKLLEGEESR  399
tr|A0A411KAS9|A0A411KAS9_DICLA      FSLETGSYQDTIGHLEEDIH----------------------------------------  162
                                    *. *: . **.* :*::: :

sp|P48674|VIME_ONCMY                ITTPMPNFSSFNLRESMLEA-------------------RPMID-NLSKKVVIKTIETRD  446
sp|P02543|VIME_PIG                  ISLPLPNFSSLNLRETNLES-------------------LPLVDTHSKRTLLIKTVETRD  451
sp|P48671|VIM1_CARAU                ISTPLPNFSSFNLRETMLEL-------------------KPNIESTFTKKVLIKTIETRD  155
tr|A0A671V9L3|A0A671V9L3_SPAAU      ITTPLASFSSLNLRGRITSLHVDITNKLFLSFLETMMDSKPHIE-TTTKKVLIKTIETRD  458
tr|A0A411KAS9|A0A411KAS9_DICLA      ------------------------------------------------------------  162

sp|P48674|VIME_ONCMY                GHVINESTQNHDDLE   461
sp|P02543|VIME_PIG                  GQVINETSQHHDDLE   466
sp|P48671|VIM1_CARAU                GQVLNESTQNHDDLE   170
tr|A0A671V9L3|A0A671V9L3_SPAAU      GQVINESTQNHDDME   473
tr|A0A411KAS9|A0A411KAS9_DICLA      ---------------   162
```

C. CLUSTAL O(1.2.4) multiple sequence alignment for S100-A1

```
tr|A0A6P6R5E7|A0A6P6R5E7_CARAU      ------------------------------------------------------------  0
tr|A0A671W834|A0A671W834_SPAAU      MAFSTLFALSLSLPLCAWVSLPLPLPSIFLGVAWLHYKYYNKLLGEPLHSSQITTKPQLL  60
tr|C1BH93|C1BH93_ONCMY              ------------------------------------------------------------  0
sp|P02639|S10A1_BOVIN               ------------------------------------------------------------  0
tr|A0A8C4I043|A0A8C4I043_DICLA      ------------------------------------------------------------  0


tr|A0A6P6R5E7|A0A6P6R5E7_CARAU      --------------MEGAIKTVVTQFL---SSARGKESLGGKNFQKLVQSQLGNILSDTD  43
tr|A0A671W834|A0A671W834_SPAAU      PLYVLHLLSAAMSELEVCMEKLILLFHKYADEDGDKKHLSKKEFKKLVETELPTFLKTQK  120
tr|C1BH93|C1BH93_ONCMY              ----------MPSDLERAMESMITVFHKYAAKEGSGNTLSRRELKDLMENELSGFLKSQK  50
sp|P02639|S10A1_BOVIN               ----------MGSELETAMETLINVFHAHSGKEGDKYKLSKKELKELLQTELSGFLDAQK  50
tr|A0A8C4I043|A0A8C4I043_DICLA      ----------MPSQLEGAMDALITVFYNYSGNDGDKYKLNKGELKELLNSELTDFLTSQK  50
                                                  :* .:. ::  *     .  .   *.  :::.*::.:*  :*   .

tr|A0A6P6R5E7|A0A6P6R5E7_CARAU      SSSAVKDMMKGLDDNQDGKVGFQEYLMLVGYLANSLSEQKAQSSAAGGP  92
tr|A0A671W834|A0A671W834_SPAAU      NPKAVECIMKDLDTNKDDKLSFEEFLPLVAGLSMACDKCYNLQQKHCKK  169
tr|C1BH93|C1BH93_ONCMY              DPATVDKIMKDLDSNGGGEVNFEEFVSLVVGLSIACEQCYQMHKKKMGK  99
sp|P02639|S10A1_BOVIN               DADAVDKVMKELDENGDGEVDFQEYVVLVAALTVACNNFFWENS-----  94
tr|A0A8C4I043|A0A8C4I043_DICLA      DPMLVEKIMNDLDSNKDNEVDFNEFVVLVAALTVACNDFFQEQKKKNK-  98
                                    .   *. :*: ** * ..::.*:*:: **  *: : ..     .
```

D. CLUSTAL O(1.2.4) multiple sequence alignment for glial fibrillary acidic protein (GFAP).

```
tr|A0A671WZT8|A0A671WZT8_SPAAU      MSSSPERMSSYRRHFEGNLAASSTYQLRVSSPSPARRESRPRSVSYTRGGGTMVRRASSK  60
sp|Q28115|GFAP_BOVIN                -MERRRVTSATRR-----------SYVSS---SEMVVGGR-----R----------LGPG  30
tr|A0A8C7NQ31|A0A8C7NQ31_ONCMY      ------------RERGRERKARRHLYL--SGRAQTIPGTHPRHMTH----------SSTI  36
tr|A0A8P4G1A2|A0A8P4G1A2_DICLA      -MEGQRYQSSYRKRYGPQGSSSTGVRIGSHSSSRLSWHGTPRNLTH----------SSPI  49
sp|P48677|GFAP_CARAU                ------------------------------------------------------------  0

tr|A0A671WZT8|A0A671WZT8_SPAAU      AGLTSSVSVGT-LCLGLETKLDLDAAAAENRAFMVTRSSERQEMVVLNDRLAVYIEKVRS  119
sp|Q28115|GFAP_BOVIN                TRLSLAR-----MPPPLPARVDFSLAGALNSGFKETRASERAEMMELNDRFASYIEKVRF  85
tr|A0A8C7NQ31|A0A8C7NQ31_ONCMY      SRLSLGSAGGALLLGTPGNRLDFSADSLLKAQYRETRTNEKVEMMGLNDRFASFIEKVRF  96
tr|A0A8P4G1A2|A0A8P4G1A2_DICLA      SRLSLGSTNTALLLGSPGDRLDFSADTLMKAQYKETRTNEKMEMMGLNDRFASYIEKVRL  109
sp|P48677|GFAP_CARAU                --------------------------------------REVDRVMGLNDRFASYIEKVRF  22
                                                                           *  .:: ****:* :*****

tr|A0A671WZT8|A0A671WZT8_SPAAU      LESKNKLLEAEIEALRSRYARPSGLRQLYESQLKDLHRVAEQMRVQRDTSLAAKEAMFGQ  179
sp|Q28115|GFAP_BOVIN                LEQQNKALAAELNQLRAK--EPTKLADVYQAELRELRLRLDQLTANSARLEVERDNLAQD  143
tr|A0A8C7NQ31|A0A8C7NQ31_ONCMY      LEQQNTVLVTELTQLRGK--EPSRLGDIFQEELRELRRQVDGLSAGKARLEIERDNMAAD  154
tr|A0A8P4G1A2|A0A8P4G1A2_DICLA      LEQQNKMLVAELNQLKGK--EPSRLGDIYQEELRELRRQVDGLTAGKARLEIERDNLATD  167
sp|P48677|GFAP_CARAU                LEQQNKMLVAELNQLRGK--EPSRLGDIYQEELRELRRQVDGLNAGKARLEIERDNLASD  80
                                    **.:*. * :*:  *:.:  .*: * :::: :*::*:   : : .        :: :  :

tr|A0A671WZT8|A0A671WZT8_SPAAU      LDSLKAKYEAAVVARKETEQDIEALRPDVDRATSARIHLEKRLEHLEVELAFLQRVHKEE  239
sp|Q28115|GFAP_BOVIN                LGTLRQKLQDETNQRLEAENNLAAYRQEADEATLARLDLERKIESLEEEIRFLRKIHEEE  203
tr|A0A8C7NQ31|A0A8C7NQ31_ONCMY      VATLKQRLQDEMVLRQDAESNLNAFRQDVDEASLNRVQLERKIDALQDEIAFLKKIHEEE  214
tr|A0A8P4G1A2|A0A8P4G1A2_DICLA      VATLKQRLQEEMGLRQDAEGNLNAFRQDVDEASLNRVQLERKIDALQDEINFLKKTHDEE  227
sp|P48677|GFAP_CARAU                LATLKQRLQEENALRQEAENNLNTFRQDVDEAALNRVQLERKIDALQDEISFLRKVHEEE  140
                                    : :*: : :     * ::* :: : * :.*.*:  *:.**:::: *: *: **:: *.**

tr|A0A671WZT8|A0A671WZT8_SPAAU      IEELMQQIYAAASKVDLTFGLPDLSTALKQIQSQYDSIAAKNLKEMDAWYASKFQDLSSA  299
sp|Q28115|GFAP_BOVIN                VRELQEQLAQQQVHVEMDVAKPDLTAALREIRTQYEAVASSNMHEAEEWYRSKFADLNDA  263
tr|A0A8C7NQ31|A0A8C7NQ31_ONCMY      LRELQEQLMAQQVHVDVDVSKPDLTAALRDIRVQYESVASSNIQETEEWYRSKFADLTDA  274
tr|A0A8P4G1A2|A0A8P4G1A2_DICLA      LREFQEQIMAQQVHVDLDVSKPDLTAALRDIRVQYETMATSNMQETEEWYRSKFADLTDA  287
sp|P48677|GFAP_CARAU                MRQLQEQLIAQQVHVDLDVSKPDLTTALKEIRAQFEAMATSNMQETEEWYRSKFADLTDA  200
                                    :.:: :*:     :*:: .. ***::**::*: *:::*:.*::* : ** *** **..*

tr|A0A671WZT8|A0A671WZT8_SPAAU      STKHAQSVRTLREEIAGYRKNILDKERELDAVKTRNEYLVNQIRDTVEKHKMEEEDLQER  359
sp|Q28115|GFAP_BOVIN                AARNAELLRQAKHEANDYRRQLQALTCDLESLRGTNESLERQMREQEERHAREAASYQEA  323
tr|A0A8C7NQ31|A0A8C7NQ31_ONCMY      ATRNADALRLAKQEGNEYRRQLQAMTCDVEALRGTNESLEQQLREMEDRFSVETAGYQDM  334
tr|A0A8P4G1A2|A0A8P4G1A2_DICLA      ASRNAEALRQAKQEANEYRRQIQVVTCDLEALRGTNESLERQLREMEDRFSMDTTGYQDT  347
sp|P48677|GFAP_CARAU                AGRNAEALRQAKQEANEYRRQIQGLTCDLESLRGSNESLERQLREMEERFAIETAGYQDT  260
                                    : ::*: :*  :.*   **:::     :::::  ** * .*:*:  ::.  :  . *:

tr|A0A671WZT8|A0A671WZT8_SPAAU      MEANKLDLKVTKEKIALLLREHQDLLNVKMALEIEITTYRKLIEGEDSRLSTTVQNLSLT  419
sp|Q28115|GFAP_BOVIN                LARLEEEGQSLKDEMARHLQEYQDLLNVKLALDIEIATYRKLLEGEENRITIPVQTFSNL  383
tr|A0A8C7NQ31|A0A8C7NQ31_ONCMY      VGHLEEEIQTLKEGMARHLQEYQDLLNVKLALDIEIATYRKLLEGEESRITVPMQSFSNL  394
tr|A0A8P4G1A2|A0A8P4G1A2_DICLA      VSHLEEEIQGLKEEMARHLQEYQDLLNVKLALDIEIATYRKLLEGEESRITIPTECFPLS  407
sp|P48677|GFAP_CARAU                VARLEDEIQMLKEEMARHLQEYQDLLNVKLALDIEIATYRKLLEGEESRITVPVQNFTNL  320
                                    :   : : :  *: :*  *:*:*******:**:***:*****:***:.*::   : :

tr|A0A671WZT8|A0A671WZT8_SPAAU      GGLQLTTSVSLCAASASDSSATAAALKLNEARDESTISRAEAASEEQSIEMSERKTVLIR  479
sp|Q28115|GFAP_BOVIN                QIRET--SLD--TKSVS-----EG---------------------------HLKRNIVVK  407
tr|A0A8C7NQ31|A0A8C7NQ31_ONCMY      QFRET--SMD--TKFSP-----EA---------------------------HVKRSIIVR  418
tr|A0A8P4G1A2|A0A8P4G1A2_DICLA      PSTHPSETNL--DTKTP-----EA---------------------------HVKRSILVR  433
sp|P48677|GFAP_CARAU                QFRDT--SLD--TKLTP-----EA---------------------------HVKRSIVVR  344
                                       .   :               .                             ::.::::

tr|A0A671WZT8|A0A671WZT8_SPAAU      TVKTDEDKYES--DTQTRTIIISGAADDTEEE        509
sp|Q28115|GFAP_BOVIN                TVEMRDGEVIKESKQEHKDVM-----------        428
tr|A0A8C7NQ31|A0A8C7NQ31_ONCMY      TVETRDGEVCRTS-------------------        431
tr|A0A8P4G1A2|A0A8P4G1A2_DICLA      TVETRDGEIIKESTTEHKDLP-----------        454
sp|P48677|GFAP_CARAU                TVETRDGEIIKESTTERKDLP-----------        365

                                    **:  :.:
```

E. CLUSTAL O(1.2.4) multiple sequence alignment for desmin.

```
tr|A0A6P6PJ00|A0A6P6PJ00_CARAU      ------------------------------------------------------------  0
sp|P17661|DESM_HUMAN                MSQAY-SSSQRVSSYRRTFGGAPGFPLGSPLSSPVFPRAGFGSKGSSSSVTSRVYQVSRT  59
tr|A0A8C4I534|A0A8C4I534_DICLA      -MASYSSSAQSASSYRRHFGHGTYAS---PSLN-----------------RSLVYEVTRS  39
tr|Q8UWF1|Q8UWF1_ONCMY              -----------ISSYRRTFGSGIGST---PGMSSMFSHGGRGSS-GSAHMSSRVYEMTKS  45
tr|A0A2R2YUL3|A0A2R2YUL3_SPAAU      MSKSYSSSAQSASSYRRTFGSGVGST---PMSSYYSSGAGGRSS-SSASMSSRVYEVKS-  55


tr|A0A6P6PJ00|A0A6P6PJ00_CARAU      MRRAHWQCGN----------------ATAGSRDKAHY-------SHF-CERT-DRAHDAH  35
sp|P17661|DESM_HUMAN                SGGAGG-LGSLRAS----RLGTTRTPSSYGAGELLDFSLADAVNQEFLTTRTNEKVELQE  114
tr|A0A8C4I534|A0A8C4I534_DICLA      SATPTYRVSSGYYGKPLAASRASVGRSYAGMGETLDFSLADALNQEFLTTRTNEKVELQH  99
tr|Q8UWF1|Q8UWF1_ONCMY              SARPSYSSGSIRSSS-GGAM-----RSYAGMGEKLDFNLADATNRDFLDTRTNEKAELQH  99
tr|A0A2R2YUL3|A0A2R2YUL3_SPAAU      SSLPSYSSYRVSSGA-GGAGYGASTAIRTYSGEKLDFNLADAMNQDFLNTRTNEKAELQH  114
                                                                :  .:        .*   ** ::..  .

tr|A0A6P6PJ00|A0A6P6PJ00_CARAU      TVFSYSPVATKVRSLEQQNKLLETEIEALKNRYLKPTGLRLLYEEQLQELKRLAEQMRIQ  95
sp|P17661|DESM_HUMAN                LNDRFANYIEKVRFLEQQNAALAAEVNRLKGRE--PTRVAELYEEELRELRRQVEVLTNQ  172
tr|A0A8C4I534|A0A8C4I534_DICLA      LNDRFASYIEKVRFLEQQNQVLAVEVERLRGRE--PTRIADLYEEEMSELRRQVEILTNQ  157
tr|Q8UWF1|Q8UWF1_ONCMY              LNDRFASYIEKVRFLEQQNATLVVEIERLRGHE--PTRVAEMYEEEMRELRRQVDGMSND  157
tr|A0A2R2YUL3|A0A2R2YUL3_SPAAU      LNDRFASYIEKVRFLEQQNAALTVEIEKLRGRE-GPGRVAEMYEEEMRELRRQIESHSNQ  173
                                        ::    *** *****  * .*:: *:.:   *  :  :***:: **:*  :    :

tr|A0A6P6PJ00|A0A6P6PJ00_CARAU      RDLAIAAKDAMAGQLEMIKVKYEEAVEMRKKAELDIEAFRPDVDAATAARIALAKQLENL  155
sp|P17661|DESM_HUMAN                RARVDVERDNLLDDLQRLKAKLQEEIQLKEEAENNLAAFRADVDAATLARIDLERRIESL  232
tr|A0A8C4I534|A0A8C4I534_DICLA      RSRVEVERDNLADDLDKLKLRLQEEILQKEDAENNLAAFRADVDAATLARLDLERRIETL  217
tr|Q8UWF1|Q8UWF1_ONCMY              RARMEVERDNLADDLQKLKLRLQEVIHQREEAENNLSAFRADVDSATLARLDLERRIESL  217
tr|A0A2R2YUL3|A0A2R2YUL3_SPAAU      RARVEVERDNLADDLQKLKLRLQEEIHQKEEAENNLSAFRADVDNATLARLDLERRIESL  233
                                    *    . :* : .:*: :* : :* :  ::.** :: *** *** ** **: * :::*.*

tr|A0A6P6PJ00|A0A6P6PJ00_CARAU      EVELEFLRRVHKEEIEELMKQIYAAHATAADAYSLPDLSSAIKQIQLQYDDIAAKNLQEM  215
sp|P17661|DESM_HUMAN                NEEIAFLKKVHEEEIRELQAQLQEQQVQVEMDMSKPDLTAALRDIRAQYETIAAKNISEA  292
tr|A0A8C4I534|A0A8C4I534_DICLA      QEEIAFLKKIHEEEIRELQSQMQETQVQIQMDMSKPDLTAALRDIRAQYEGIAAKNIAEA  277
tr|Q8UWF1|Q8UWF1_ONCMY              QEEITFLKKIHEEEIHELTSQMQETSVQVQMDMSKPDLTVALRDIRMQYEGIAAKNISEA  277
tr|A0A2R2YUL3|A0A2R2YUL3_SPAAU      QEEIGFLKKIHEEEIRELQSQMQESQVQIQMDMSKPDLTAALRDIRMQYEGIAAKNISEA  293
                                    : *: **:::*:***.**  *:     .      * ***: *:::*: **: *****: *

tr|A0A6P6PJ00|A0A6P6PJ00_CARAU      DSWYKSKFDDLNNKSSKHVDKVRSVREEIVTAKKDIQNKERDLDSLKTKNEALEAQIRET  275
sp|P17661|DESM_HUMAN                EEWYKSKVSDLTQAANKNNDALRQAKQEMMEYRHQIQSYTCEIDALKGTNDSLMRQMREL  352
tr|A0A8C4I534|A0A8C4I534_DICLA      EDWYKSKVSDLNQAVSKNNEALKQARQETMEFRHQIQSYTCEIDSLKGTNESLMRQMRDM  337
tr|Q8UWF1|Q8UWF1_ONCMY              EDWYKSKVSDLNQAVNKNNDALRQAKQESMEFRHQIQSYTCEIDSLKGTNESLLRQMREM  337
tr|A0A2R2YUL3|A0A2R2YUL3_SPAAU      EEWYKSKVSDLNQAVNKNNDALRQAKQESMEYRHQIQSYTCEIDSLKGTNESLLRQMRDM  353
                                    :.*****..**.:  .*: : ::..::* :  :::**.   ::*:** .*::*  *:*:

tr|A0A6P6PJ00|A0A6P6PJ00_CARAU      QEKYRKELEELQARIEALQLELKSSKQRTAMLLREYQDLLNVKMALEIEITTYRKLIEGE  335
sp|P17661|DESM_HUMAN                EDRFASEASGYQDNIARLEEEIRHLKDEMARHLREYQDLLNVKMALDVEIATYRKLLEGE  412
tr|A0A8C4I534|A0A8C4I534_DICLA      EDRHGVEAGRFQDNIARLEAEIANMKDEMARHLREYQDLLNVKMALDVEIATYRKLLEGE  397
tr|Q8UWF1|Q8UWF1_ONCMY              EDRLGNEAGGYQDSVTRLEAEIAKMKDEMARHLREYQDLLNVKMALDIEIATYRKLLEGE  397
tr|A0A2R2YUL3|A0A2R2YUL3_SPAAU      EDRMGREASGFQDTIARLEADIAKMKDDMARHLREYQDLLNVKMALDIEIATYRKLLEGE  413
                                    :::   *    *  :  *: ::    *:  *  *************::**:*****:***

tr|A0A6P6PJ00|A0A6P6PJ00_CARAU      DSRITSVMQSMQTMSLMSGSISVHSAGAAGVAGIVDKVGGGPDGGAASGLGGGNGGGLLN  395
sp|P17661|DESM_HUMAN                ESRINLP-----------------------I----------------------------  420
tr|A0A8C4I534|A0A8C4I534_DICLA      ESRITLP-----------------------V----------------------------  405
tr|Q8UWF1|Q8UWF1_ONCMY              ESRITVSGSKSSH----SGSHSA--------A----------------------------  417
tr|A0A2R2YUL3|A0A2R2YUL3_SPAAU      ESRITTTAP-------------V--------Q----------------------------  424
                                    :***.

tr|A0A6P6PJ00|A0A6P6PJ00_CARAU      GITSGVGGPGTTVDYTQEQAVEKTERKTVLIRTVKTEDDTLESNTQEKSYSISGAADDEE  455
sp|P17661|DESM_HUMAN                QTYSALNFRETSPE---QRGSEVHTKKTVMIKTIETRDGEVVSEATQQQHEVL-------  470
tr|A0A8C4I534|A0A8C4I534_DICLA      QSYSTLSFRETSPEHQ-QRASEMHSKKTVLIKTIETRDGEVVSESTQHQQDIM-------  457
tr|Q8UWF1|Q8UWF1_ONCMY              SLYSTVGFRETSPDV--GRSAEVHSKKTVLIKTIETRDGEVVSE----------------  459
tr|A0A2R2YUL3|A0A2R2YUL3_SPAAU      SAYSSIGFRETSPESQHQRSSEVHSKKTVLIKTIETRDGEVVSESTQHQQDIM-------  477
                                       * :.   *: :    :. *   :***:*:*::*.*. : *:
```
